# Disentangling neuroimmune landscapes across peripheral activation paradigms resolves divergent glial state programs

**DOI:** 10.21203/rs.3.rs-8844780/v2

**Published:** 2026-03-11

**Authors:** Mahesh Chandra Kodali, Zhengjun Wang, Geng Lin, Francesca-Fang Liao

**Affiliations:** 1. Department of Neurology, Harvard Medical School, Boston, MA, 02115, USA; 2. Department of Neurology, Massachusetts General Hospital, Charlestown, MA, 02129, USA; 3. Department of Pharmacology, Addiction Science and Toxicology, College of Medicine, University of Tennessee Health Science Center, Memphis, TN – 38103, USA; 4. Integrated Biomedical Sciences Program, Molecular and Systems Pharmacology Track, College of Graduate Health Sciences, University of Tennessee Health Science Center, Memphis, TN – 38163, USA

**Keywords:** Microglia, Repeated Low Dose LPS, Neuroinflammation, Lipopolysaccharide, CNS myeloid cells

## Abstract

Lipopolysaccharide (LPS), a gram-negative bacterial cell-wall component, is a well-characterized immunostimulant acting via Toll-like receptor 4 (TLR4) and is widely used to model systemic inflammation–to–brain immune signaling. A single intraperitoneal high dose evokes a robust peripheral inflammatory state that is rapidly relayed to the central nervous system (CNS), resulting in profound neuroinflammation. By contrast, repeated low-dose LPS engages innate immune memory and has been associated with neuroprotective effects. Here we sought to comprehensively characterize the CNS-specific effects of the repeated LPS regimen relative to a neurotoxic single high-dose challenge. High-dose LPS induced robust forebrain inflammatory cytokine expression, disrupted homeostatic microglial and astrocytic marker programs, and produced transcriptomic signatures enriched for NF-κB signaling and apoptosis. In striking contrast, repeated low-dose LPS preserved homeostatic glial marker expression while increasing IBA1/F4/80-positive microglial signal across forebrain regions without a parallel increase in inflammatory cytokine transcripts, demonstrating a dissociation between gliosis marker intensity and inflammatory pathway activation. Whole-forebrain RNA-seq demonstrated selective enrichment of phagocytosis-related pathways under the repeated regimen in the absence of pro-inflammatory transcriptional amplification. Flow cytometry revealed an expansion of CD45^high^ CD11b^+^ myeloid cells expressing the phagocytic marker CD206 following repeated low-dose LPS. Cell-type–resolved transcriptional profiling showed that this CD45^high^ CD11b^+^ subset preferentially upregulated phagocytic programs while lacking prominent pro-inflammatory and apoptotic pathway activation. In parallel, astrocytes maintained homeostatic gene expression without enrichment of neurotoxic inflammatory signatures. Together, these findings delineate how distinct systemic LPS dosing paradigms differentially shape glial transcriptional and phenotypic responses in the CNS.

## Introduction:

Lipopolysaccharide (LPS), an outer cell-wall component of gram-negative bacteria, is a canonical agonist of Toll-like receptor 4 (TLR4), and has been widely employed as an experimental tool to study systemic inflammation and its effects on the central nervous system (CNS)(1, 2). A single high intraperitoneal dose induces a strong peripheral cytokine response that rapidly reaches the brain(3), leading to activation of microglia and astrocytes, disruption of glial homeostasis, and subsequent neuronal injury(1, 4-6). This acute neuroinflammatory state has been used extensively to model the neuroinflammatory phase of neurodegenerative conditions(7) and as such has been instrumental in helping to understand the crosstalk between peripheral immune activation and the CNS.

Mechanistically, peripheral endotoxin exposure can trigger rapid CNS innate responses through neurovascular and border interfaces rather than requiring direct microbial invasion of the parenchyma. In rodents, peripheral LPS induces cytokine transcripts and cytokine-responsive genes in barrier-associated structures including the choroid plexus, meninges, and circumventricular organs—sites positioned to sense circulating inflammatory cues and relay them to brain circuits (1, 8). Consistent with this interface model, TLR4 expression and inducibility across the brain and neurovascular compartments have been implicated in translating systemic endotoxin signals into CNS innate activation programs(2, 9). Critically, these pathways can be engaged either as a single acute inflammatory insult or as repeated subthreshold exposures, and the temporal pattern of stimulation is now recognized as a major determinant of the resulting CNS glial state.

In the setting of repeated exposures, brain-resident myeloid cells can develop a form of immune memory in which subsequent inflammatory outputs are either amplified (“training”) or dampened (“tolerance”), with both states supported by durable chromatin remodeling in (10-12). In contrast to the acute neurotoxic response elicited by a single high-dose LPS challenge, repeated administration of low doses of LPS elicits a distinct form of innate immune memory(10, 13, 14). Depending on dosing frequency, innate immune cells in the CNS, including microglia, undergo transcriptional and epigenetic reprogramming that results in either a trained state with amplified inflammatory responses or a tolerant state characterized by dampened cytokine induction and increased phagocytic competence(10, 14). Immune training is found to exacerbate amyloid-β deposition, whereas immune tolerance alleviates it, with H3K4me1-mediated enhancer remodeling identified as a mechanistic underpinning(10, 11). Extending these findings to humans, neuroimaging studies show that repeated systemic LPS exposure reprograms cerebral immune responses, with subsequent inflammatory challenges eliciting attenuated neuroinflammatory signal(15). These observations highlight that repeated low-dose LPS may offer a window into protective glial reprogramming.

Several recent studies have extended this concept(16). Repeated low-dose LPS regimens (0.5–1 mg/kg for 3–5 days) have been shown to reduce brain injury through endothelial MyD88–CXCL10 signalling(17), limit microglial COX-2 induction and behavioural deficits(18), and induce region-specific microglial tolerance signatures(12). Nemeth et al identified IL-1R1-dependent microglia–vascular interactions following repeated systemic LPS as contributors to neuroprotection(9). Together these findings emphasize that repeated low-dose paradigms can promote CNS protection, but the precise cellular and molecular pathways engaged in different glial subsets remain to be fully defined.

Microglia and astrocytes are highly heterogeneous populations whose activation states critically shape outcomes in neurodegenerative disease. While the majority of microglia are CD45 ^low-mid^ TMEM119^+^ homeostatic cells, a smaller CD45 ^high^ CD11b^+^ population with enhanced phagocytic capacity has been described(19). Astrocytes similarly polarize towards neurotoxic A1 or protective phenotypes depending on the surrounding immune milieu(20). However, how repeated low-dose LPS tolerance regimens impact these distinct glial compartments, and how they differ from the acute neurotoxic response to high-dose LPS, remains poorly understood. We thus aimed to comprehensively characterize the CNS-specific effects of repeated low-dose LPS administration in contrast to the neurotoxic effects of a single high-dose LPS treatment.

Here, we sought to define how repeated low-dose LPS, a tolerance-inducing regimen, alters glial states in the CNS compared to the neurotoxic response elicited by a single high dose. Using bulk forebrain transcriptomics together with flow cytometry and RNA-sequencing of sorted glial populations, we comprehensively mapped the molecular pathways engaged under each LPS dosing paradigm.

## Methods

### Animals:

C57BL/6 mice were obtained from Jackson Laboratories, housed and bred in well-ventilated cages under standard laboratory conditions on 12:12 hour light-dark cycle with food and water ad libitum. Both male and female mice aged between eight to twelve weeks were used unless otherwise mentioned. All animal experimental procedures were conducted in accordance with the animal care standards of the National Institute of Health and were approved by the Institutional Animal Care and Use Committee (IACUC) of the University of Tennessee Health Science Center.

### Lipopolysaccharide (LPS) treatment:

Mice were randomized into experimental groups and received intraperitoneal injection of either endotoxin-free phosphate buffered saline (PBS) or a single high dose (LPS_High) of LPS from *E. coli* O55:B5 (Sigma-Aldrich L2880) dissolved and diluted in endotoxin-free PBS (10 mg kg^−1^ body weight) or repeated low-dose (LPS_Low_Repeated) once daily for four days (0.5 mg kg^−1^ body weight) or a single low dose (LPS_Low) (0.5 mg kg^−1^ body weight) (21-24). Mice were sacrificed at 24 hours post-injection for single dose, and post 24 hours after last injection for the repeated low dose regimen.

### Immunofluorescent staining:

Following transcardial perfusion, brains were rapidly harvested and immersion-fixed in 4% paraformaldehyde prepared in PBS for 24 h. Fixed tissues were subsequently cryoprotected in 30% sucrose/PBS for 72 h, embedded in optimal cutting temperature (OCT) compound (#23-730-571, Fisher Scientific, Hampton, NH, USA), and coronally sectioned at 15 μm using a Leica CM1850 cryostat. Sections were mounted onto glass microscope slides (#12-550-15, Fisher Scientific) and dried on a slide warmer at 37 °C for 1 h. For immunofluorescence staining, tissue sections were permeabilized in 0.1% Triton X-100 in PBS for 10 min at room temperature, followed by blocking in 5% goat serum diluted in PBS for 1 h to reduce non-specific antibody binding. Primary antibodies were diluted in antibody dilution buffer (#25886-05, Electron Microscopy Sciences, Hatfield, PA, USA) and incubated with the sections overnight at 4 °C. The following primary antibodies were used: rabbit anti-Iba1 (#019-19741, Wako Laboratory Chemicals, Richmond, VA, USA; 1:1000), mouse anti-GFAP (G3893, Sigma-Aldrich, St. Louis, MO, USA; 1:500), and rat anti-F4/80 (MCA497R, Bio-Rad Laboratories, Hercules, CA, USA; 1:200). After primary antibody incubation, sections were washed three times in PBS (5 min per wash) and incubated with Alexa Fluor–conjugated secondary antibodies (Thermo Fisher Scientific, Waltham, MA, USA; 1:500) for 1 h at room temperature. Sections were then washed, counterstained with DAPI, air-dried, and coverslipped using Fluoromount-G mounting medium (#0100-01, SouthernBiotech, Birmingham, AL, USA).

### Image quantification:

Fluorescent images were acquired at a resolution of 4080 × 3072 pixels using an Olympus IX50 microscope (Olympus Corporation, Shinjuku, Tokyo, Japan). Image acquisition was restricted to anatomically defined hippocampal and neocortical regions. For each brain, approximately 50 coronal sections spanning the hippocampus (20 μm thickness) were generated, and one section from every eight sequential sections was selected, yielding a six-section series used for quantitative analysis. Prior to imaging, all sections were visually inspected to ensure preservation of hippocampal and cortical cytoarchitecture, as well as proper section flattening without folds or tissue distortion. For each animal, six to eight images per anatomical region were captured, sampling as many sections as possible from the six-section series. Exposure settings were manually optimized to avoid pixel saturation while preserving signal-to-background contrast, and identical exposure times were maintained across images acquired for the same antibody. Raw images were subsequently downsampled to 680 × 512 pixels and processed using ImageJ software (National Institutes of Health, Bethesda, MD; https://imagej.nih.gov/ij/). Images were converted to RGB stacks, color-inverted, and transformed into grayscale format. Threshold values were then uniformly applied to delineate immunoreactive signal from background. Quantification of staining was performed using integrated intensity measurements (mean gray value × area), obtained via the ROI Manager function in ImageJ. Positive immunoreactive aggregates were defined by size constraints ranging from 3 to 500 pixel² and circularity values between 0 and 1. Structures lacking discernible cellular morphology were manually excluded to eliminate false-positive signal. For each animal, the mean integrated intensity derived from six to eight images was calculated and used as the representative value for that brain.

### Brain single cell suspension:

Single cell suspensions were prepared from dissected forebrain tissue, including the prefrontal cortex and hippocampus as previously described (25). Briefly, forebrains were carefully dissected free of meninges, mechanically cut into small pieces, followed by resuspension in 2.5 ml of Hank’s balanced salt solution (HBSS) containing 50 units of activated papain (Worthington, LK003172) and 250 units of activated DNase (Worthington, LK003178), and incubated at 37°C for 30 minutes. Prechilled HBSS with 2 mM EDTA and 2% FBS was added to halt the digestion, and the cells were pelleted at 300 g for 10 minutes at 4°C. The cells were then gently triturated with a glass Pasteur pipette, followed by passing through a 70 µm filter (Miltenyi, 13098462). The cells were then pelleted at 300Xg for 10 minutes at 4°C, and resuspended in 12 ml of 22% isotonic 4°C Percoll plus (Millipore Sigma, E0414-250ML) in 1× HBSS and centrifuged with low break at 560 g at 4°C for 20 minutes. The top myelin debris was then carefully discarded, and the cells were washed with HBSS containing 2% FBS, and then finally resuspended in HBSS and stained with Live/Dead Fixable Blue Dead Cell stain (Thermo Fisher, L23105) and proceeded to immunolabelling in 200 ml of HBSS containing 2% FBS and 1:100 diluted mouse CD16-CD32 Pure MAB 2.4G2 Fc Block (BD Biosciences, 553141) for 10 minutes.

### Immunolabelling:

The cells were stained by addition of the following antibodies: 1:1000 BV421 Rat Anti-Mouse CD45 Clone 30-F11 (BD Biosciences, 563890), 1:50 Alexa Fluor^®^ 700 anti-mouse CD206 (BioLegend, 141734), 1:50 Tmem119 Monoclonal Antibody (V3RT1GOsz), PE-Cyanine7 (eBioscience, 25-6229-82), 1:800 BD Pharmingen^™^ APC-Cy^™^7 Rat Anti-CD11b Clone M1/70 (BD Biosciences, 557657), 1:200 ACSA-2 Antibody, anti-mouse, REAfinity^™^ Clone REA969 ∣ IH3-18A3 (Miltenyi Biotec, 130-116-249), 1:25 Rat anti mouse Alexa Fluor^®^ 488 F4/80 antibody Clone A3-1(Biorad, MCA497A488), Rat Anti-Human/Mouse TREM-2 PE-conjugated Monoclonal Antibody Clone 237920 (Novus Bio, FAB17291P). Antibody stained cells were then incubated on ice in the dark for 10 minutes, and then washed three times with HBSS containing 2% FBS. Finally, the cells were resuspended in HBSS containing 2% FBS, 1:100 RNase free DNase (Qiagen, 79254) and 1:500 RNasein plus RNase inhibitor (Promega, N2611).

Fluorescence-activated cell sorting (FACS): Cell sorting was performed using a BD Aria II cell sorter using the 100-micron nozzle. Cells were sorted at 4°C and collected directly into Buffer RLT-Plus (Qiagen, 1053393) containing 1:100 β-mercaptoethanol (Millipore Sigma, 444203-250ML). Appropriate single colored controls and fluorescence minus one controls (FMOs) were included and the sorting gates were setup accordingly.

### RNA sequencing (RNA-seq):

Isolated RNA sample quality was determined using High Sensitivity RNA Tapestation (Agilent Technologies Inc., California, USA) and concentration was measured using Qubit 2.0 RNA High Sensitivity assay (ThermoFisher, Massachusetts, USA). Libraries were then constructed following manufacturer’s instructions using NEBNext Ultra II RNA kit with PolyA selection for the bulk RNA samples, and using SMART-Seq^®^ v4 Ultra^®^ Low Input RNA Kit (Takara Bio USA Inc., California, USA) followed by Nextera^®^ XT DNA Library Prep Kit (Illumina, California, USA) for FAC sorted cells’ RNA. Library concentration was initially measured using a Qubit 2.0 fluorometer (Life Technologies) and then diluted to 2 ng/μl before checking insert size on an Agilent 2100 and quantifying to greater accuracy by KAPA SYBR^®^ FAST quantitative PCR (Roche, Indianapolis, USA) (library activity >2 nM). The resulting final library size was about 430bp with an insert size of about 300bp. Illumina^®^ 8-nt dual-indices were used. Equimolar pooling of libraries was performed basing on QC values and sequencing was performed on an Illumina^®^ NovaSeq 6000 (Illumina, California, USA) for the forebrain samples or Illumina^®^ HiSeq 4000 (Illumina, California, USA) for FAC sorted cell samples by Novogene Co. (Sacramento, CA). The read length configuration was 150 PE for 40 M PE reads per sample (20M in each direction).

### RNA-seq data analysis:

Transcript abundance from RNA-seq reads were quantified using Salmon (26), and gene-level counts were obtained using tximport (27), against the C57BL/6J mouse genome annotation Genome Reference Consortium Mouse Build 38 patch release 6 (GRCm38.p6), obtained from the National Center for Biotechnology Information. Subsequently, raw counts were processed with DESeq2 (28) to determine differentially expressed genes. All statistical analyses were conducted using R version 4.1. Multiple hypothesis correction was done using the Benjamini–Hochberg method, and a P-adjusted value (p.adj) of 0.05 was considered significant. R package ggplot2 (29) was used for plotting MA-plots. Heatmaps were generated after normalization of the raw counts using DESeq2, accounting for library size and removing heteroskedasticity of the counts, and finally, the values were z-scored gene wise. Z-scores were calculated and obtained on a gene-by-gene basis by subtracting the mean and then dividing by the standard deviation. In pheatmap, Z-scores are computed after the hierarchical clustering, so that it only affects the visualization. R package clusterProfiler (30) was used to determine gene ontology (GO) categories that were enriched in the significant genes from each pairwise comparison. The function enrichKEGG in clusterProfiler was used to find the KEGG pathways that were enriched from the gene sets in each of the pairwise comparisons. All significant genes (p.adj < 0.05 and LFC > 1.5) which were differentially expressed were used, and a q value cut off of 0.01 was applied. Signaling pathway impact analysis (SPIA) was done using the R package SPIA (31) to obtain the perturbation plots at each treatment comparison within in each of the cell types.

### Statistics:

All statistical analyses (excluding RNA-seq data) were conducted using GraphPad Prism v 10.5 (GraphPad Software, San Diego, CA). Data are presented as mean ± SEM. The sample size for each experiment and the statistical testing methods are reported in the figure legends.

## Results:

### Figure 1:

**Figure 1.**
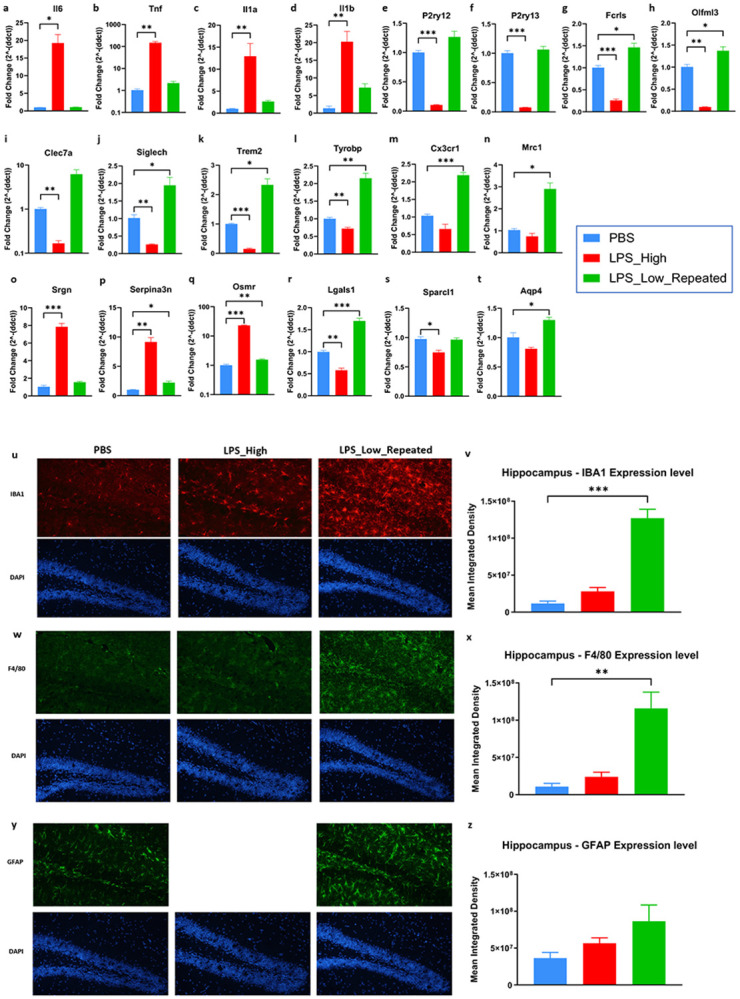
Repeated low-dose LPS preserves glial homeostasis and avoids inflammatory activation compared to high-dose LPS. Repeated low-dose LPS regimen elicits starkly contrasting effects to that of the high dose LPS in the forebrain. a–t. Gene expression analysis of pro-inflammatory cytokines and glial markers in mouse forebrain tissue in the treatment groups PBS, LPS_High, and LPS_Low_Repeated regimens. Bar graphs represent the fold change in mRNA levels of the following genes: (a) *Il6*, (b) *Tnf*, (c) *Il1a*, (d) *Il1b*, (e) *P2ry12*, (f) *P2ry13*, (g) *Fcrls*, (h) *Olfml3*, (i) *Clec7a*, (j) *Siglech*, (k) *Trem2*, (l) *Tyrobp*, (m) *Cx3cr1*, (n) *Mrc1*, (o) *Srgn*, (p) *Serpina3n*, (q) *Osmr*, (r) *Lgals1*, (s) *Sparcl1*, (t) *Aqp4*. All data are presented as mean ± SEM, One-way ANOVA, Dunnett’s post hoc test, *p < 0.05, **p < 0.01, and ***p < 0.001, (n = 6-8 mice (both male and female) in each group). u–z. Immunofluorescence analysis of glial cell markers in the hippocampus following PBS, LPS_High, and LPS_Low_Repeated regimen treatments. (u) Representative images showing IBA1 (red) staining and DAPI (blue) for nuclei. (v) Quantification of IBA1 expression levels. (w) Representative images showing F4/80 (green) staining and DAPI for nuclei. (x) Quantification of F4/80 expression levels. (y) Representative images showing GFAP (green) staining and DAPI for nuclei. (z) Quantification of GFAP expression levels. (All data are presented as mean ± SEM, One-way ANOVA, Dunnett’s post hoc test, *p < 0.05, **p < 0.01, and ***p < 0.001, (n = 6-8 mice (both male and female) in each group).

To define the CNS-specific effects of the distinct LPS paradigms, we initially quantified the expression levels of pro-inflammatory cytokine genes in the forebrain lysates collected from mice that were earlier treated with either PBS or LPS_High or LPS_Low_Repeated regimens. Gene expression analysis by qPCR revealed that the LPS_High group exhibited significant upregulation of pro-inflammatory cytokines Il6, Tnf, Il1a, and Il1b compared to the PBS control group (Fig. 1a-d), indicating a robust pro-inflammatory effect. In contrast, the repeated low-dose LPS group had cytokine expression levels close to those of the PBS control group, suggesting limited profound inflammatory activation. We also examined microglial genes *P2ry12, P2ry13, Fcrls, Olfml3, Clec7a, Siglech, Trem2, Tyrobp, Cx3cr1,* and *Mrc1* (Fig. 1e-n). In the LPS_High group, there was significant downregulation of these genes, consistent with loss of homeostatic microglial signature and function. Conversely, the LPS_Low_Repeated group has a preserved or enhanced expression of these genes compared to PBS group. Microglial genes *Olfml3, Clec7a, Tyrobp, Cx3cr1,* and *Mrc1*, are associated with phagocytic activity. Their upregulation in the LPS_Low_Repeated group is consistent with enrichment of clearance/phagocytosis-associated programs without accompanying inflammation. The LPS_High group but not LPS_Low_Repeated group showed a significant increase in the expression of *Srgn, Serpina3n,* and *Osmr* (Fig. 1o-q), which are associated with neurotoxic reactive astrocytes. Additionally, the expression of astrocyte homeostatic genes *Lgals1, Sparcl1* and *Aqp4* are decreased in the high-dose LPS group (Fig. 1r-t), indicating that high-dose LPS disrupts astrocyte homeostatic functions. In the LPS_Low_Repeated group, these gene levels indicated the maintenance or increase in expression compared to the control, suggesting that astrocyte functions related to homeostasis are promoted without inducing neurotoxic reactive astrocyte phenotype. Interestingly, the LPS_Low_Repeated group displayed increased hippocampal and cortical staining for microglial markers IBA1(Fig. 1u and 1v, Supp fig. 1a, 1b and 1g) and F4/80 (Fig. 1w and 1x, Supp fig. 1c, 1d and 1g) as well as astrocytic GFAP (Fig. 1y and 1z, Supp fig. 1e and 1f) compared to LPS_High and the PBS groups (Fig. 1u and 1v), which suggests an enhanced glial activation state albeit without an accompanying pro-inflammatory profile.

These findings suggest that glial activation under repeated low-dose LPS regimen does not necessarily correspond to an overtly inflammatory phenotype but reflects a shift toward clearance-associated and neuroprotective functions such as debris clearance while preserving CNS homeostasis.

### Figure 2:

**Figure 2.**
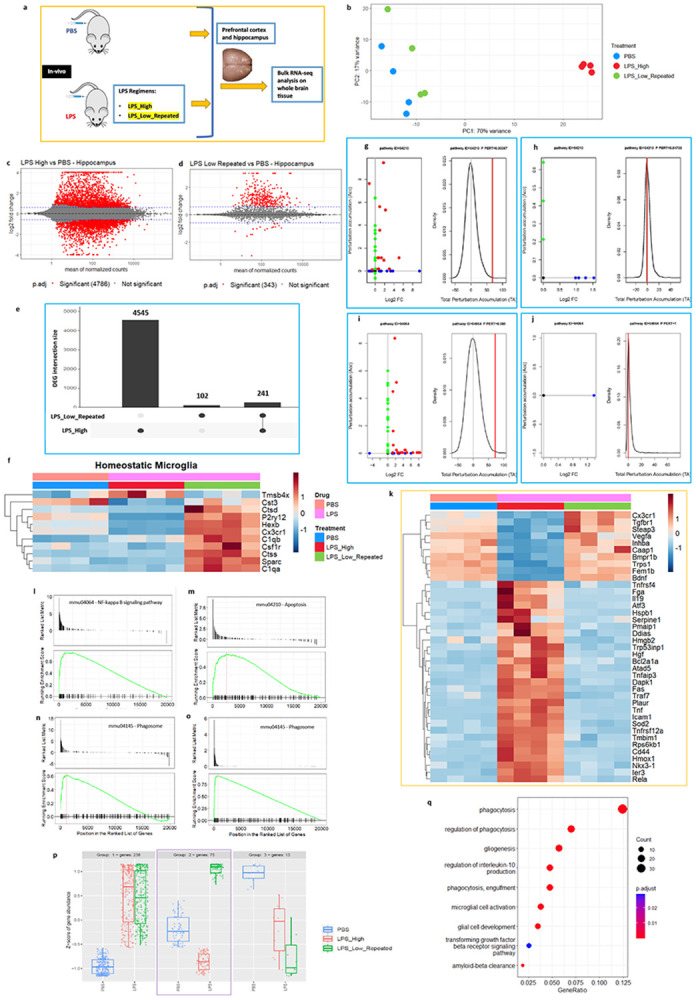
Forebrain transcriptomics reveal selective phagocytic pathway enrichment after repeated low-dose LPS. a. Scheme illustrating the experimental design - mice were injected with either LPS_High or LPS_Low_Repeated regimens or with PBS, prefrontal cortex including hippocampus were dissected and RNA was isolated followed by RNA-sequencing (n = 4 (males) in each group). b. Principal Component Analysis (PCA) plot showing the global transcriptomic differences across the treatment groups PBS, LPS_High, and LPS_Low_Repeated. c-d. MA plots of the pairwise comparisons of LPS treatments (c) LPS_High and (d) LPS_Low_Repeated as compared to PBS. Log fold changes (LFCs) are plotted against the mean of normalized counts to determine the variance between two treatments in terms of gene expression. Red nodes on the graph represent statistically significant data points i.e. p.adj < 0.05 and LFC > 1.5. Gray nodes are data points that are not statistically significant. Numerical values in parentheses for the significant legend indicate the number of genes that meet the prior condition. Dashed lines indicate the cutoff LFC values. e. Upset plot showing the intersection of differentially expressed genes (DEGs) in each LPS_High and LPS_Low_Repeated treatment groups as compared to PBS. The vertical bars represent the size of each gene set or intersection. The largest set corresponds to 4545 genes unique to the LPS_High group. 102 genes are unique to the LPS_Low_Repeated group. The intersecting set contains 241 genes that are shared between both LPS_High and LPS_Low_Repeated groups. The connected dots below the bars indicate the groups being compared, where filled circles represent the presence of genes in the respective group(s). f. Heatmap showing the expression of homeostatic microglial genes in LPS_High (red) and LPS_Low_Repeated (green) regimens compared to PBS (blue). g-j. Perturbation plots for the apoptosis pathway (Kyoto Encyclopedia of Genes and Genomes (KEGG) ID mmu:04210) (g) in LPS high compared to PBS (h) in LPS_Low_Repeated compared to PBS, and for NF-κB pathway (KEGG ID mmu:04064) (i) in LPS_High compared to PBS (j) in LPS_Low_Repeated compared to PBS. The perturbation of all genes in the pathway are depicted as a function of the log2 fold changes (left panel). Non differentially expressed genes are assigned 0 log2 fold-change. The null distribution of the net accumulated perturbation is also shown as a grey vertical line (right panel). The observed total accumulation (tA) with the actual data is shown as a red vertical line (right panel). k. Heatmap showing the differentially expressed significant genes (p.adj < 0.05 and LFC > 1.5) belonging to apoptosis pathway (KEGG ID mmu:04210), in LPS_High (red) and LPS_Low_Repeated (green) regimens compared to PBS (blue). l-o. Enrichment plots for selected KEGG pathways from GSEA analysis. (l) NF-kappa B signaling pathway (KEGG ID mmu04064) in the treatment LPS_High compared to PBS (m) Apoptosis pathway (KEGG ID mmu04210) in the treatment LPS_High compared to PBS. Phagosome pathway (KEGG ID mmu04145) (n) in the treatment LPS_High compared to PBS (o) in the treatment LPS_Low_Repeated compared to PBS. For all panels, the upper plot shows the ranked list metric of genes, and the lower plot shows the running enrichment score of the pathway. The x-axis represents the position in the ranked list of genes, while the green line represents the running enrichment score. p. Box plots showing the Z-score of gene abundances for three clusters of gene expression patterns across the treatments PBS, LPS_High, and LPS_Low_Repeated. q. Gene ontology (GO) enrichment dot plot showing the number of genes affected in the top enriched GO terms in the selected genes from group 2 cluster, from the LPS_Low_Repeated as compared to PBS.

To gain further insight into the effects of distinct LPS regimens on the whole transcriptome level, we performed RNA-sequencing of the forebrain (including the prefrontal cortex and hippocampus) lysates (Fig. 2a). This allowed us to capture a comprehensive view of the molecular responses induced by the two distinct LPS regimens. Analysis of the RNA-seq data followed by the principal component analysis (PCA) showed a clear separation among these treatment groups (Fig. 2b). The scatterplot matrix shows the expression levels of the pairwise comparisons of the LPS treatments to PBS control (Supp fig. 2a). Differential gene expression analyses revealed that LPS_High triggers a large number of differentially expressed genes (DEGs), with 4786 being significant (p.adj < 0.05 and LFC > 1.5) (Fig. 2c). In contrast, the LPS_Low_Repeated treatment resulted in 343 significant DEGs (Fig. 2d). The intersection between both treatments revealed 241 shared genes, suggesting some common responses to both LPS treatments (Fig. 2e, Supp fig. 2b and 2c). We then looked at the expression of homeostatic microglial genes (Fig. 2f) which showed that LPS_Low_Repeated treatment largely preserved the expression of these genes, which were significantly downregulated in the LPS_High group.

To get further insights into the pathways affected, we performed functional analysis on the DEG sets using signalling pathway impact analysis (SPIA) and gene set enrichment analysis (GSEA). SPIA revealed strong activation of the apoptosis pathway (KEGG ID mmu04210) and NF-κB signaling pathway (KEGG ID mmu04064) in the LPS_High group (Figs. 2g-2j). For the apoptosis pathway (Figs. 2g), there is a significant activation in the LPS_High group with a clear perturbation, as shown by the total accumulation (tA) (marked deviation in the density plot) and the positive log2 fold changes for several genes involved in this pathway. In contrast, the LPS_Low_Repeated group shows minimal to no perturbation (Fig 2h), suggesting that LPS_Low_Repeated does not activate the apoptosis pathway. The heatmap shows that apoptosis-related genes remain unchanged in the LPS_Low_Repeated group compared to PBS (Fig. 2k). Additionally, the NF-κB signaling pathway (Figs. 2i and 2j) is notably activated in the LPS_High group, with many genes showing significant positive log2 fold changes and a strong perturbation. In the LPS_Low_Repeated group, no significant perturbation accumulation or gene activation is observed, indicating that NF-κB signaling is not profoundly activated after repeated low doses of LPS. In contrast, Fc gamma R-mediated phagocytosis pathway (Supplementary Figs. 2d and 2e), shows significant activation in the LPS_Low_Repeated treatment. However, the LPS_High group shows a weaker response in this pathway, with fewer genes significantly perturbed and a lower total accumulation score. GSEA also identified significant enrichment of NF-κB signaling pathway (Fig. 2l) and apoptosis pathways (Fig. 2m) only in the LPS_High group, aligning with the strong inflammatory and cell death responses observed in this treatment. In contrast, the LPS_Low_Repeated group showed no enrichment for these pathways. Notably, the phagosome pathway was enriched in both LPS_High and LPS_Low_Repeated treatments (Figs. 2n and 2o). This supports the perturbation analyses results, where LPS_Low_Repeated revealed the exclusive activation of phagocytosis pathway without evoking inflammatory and cell-death pathways.

We next explored the gene expression patterns across the two treatments, which revealed three distinct groups of clusters varying in the expression patterns (Fig. 2p). Group 2 stood out as it exhibited contrasting gene expression in the LPS_Low_Repeated group compared to LPS_High. In this group, genes that were downregulated in LPS_High were upregulated in LPS_Low_Repeated, indicating a contrasting effect of LPS treatment regimes. We then performed gene ontology (GO) enrichment analysis on the Group 2 genes, which revealed that these genes are primarily involved in processes related to phagocytosis, including regulation of phagocytosis, and amyloid-beta clearance (Fig. 2q). Additionally, the expression of reactive astrocyte genes (Supp fig. 2f) were significantly upregulated in the LPS_High group but not in the LPS_Low_Repeated group suggesting that neurotoxic reactive astrogliosis is not induced in LPS_Low_Repeated group.

In summary, while the LPS_High treatment triggers strong activation apoptosis and NF-κB signaling, the LPS_Low_Repeated treatment avoids triggering these pathways and instead uniquely activates the phagocytosis pathway, indicating a shift towards a more regulated response focused on debris clearance without triggering apoptosis or inflammatory cascades.

### Figure 3:

**Figure 3.**
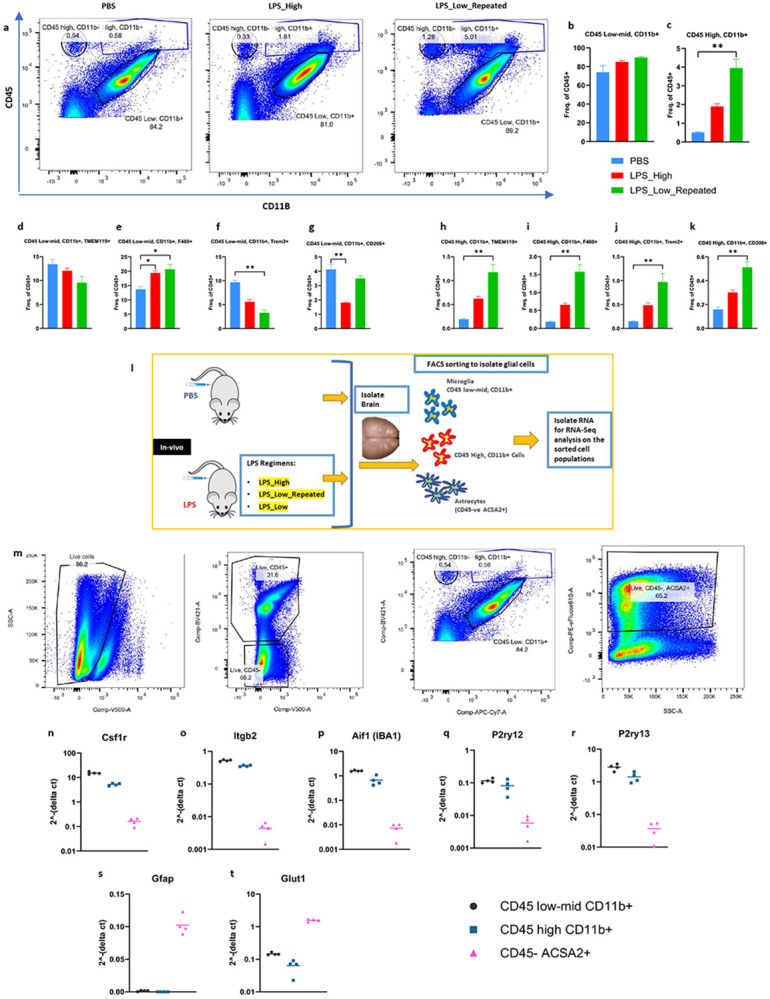
Repeated low-dose LPS leads to the expansion of CD45 ^high^ CD11b^+^ population in the brain a. Representative flow cytometry plots showing the gating strategy used to identify CD45 high CD11b+ population cells in the PBS, LPS_High and LPS_Low_Repeated treatment groups. b-k. Frequency of (b) CD45 low-mid CD11b+ cells (c) CD45 high CD11b+ cells (d) CD45 low-mid CD11b+ TMEM119+ cells (e) CD45 low-mid CD11b+ F4/80+ cells (f) CD45 low-mid CD11b+ TREM2+ cells (g) CD45 low-mid CD11b+ CD206+ cells (h) CD45 high CD11b+ TMEM119+ (i) CD45 high CD11b+ F4/80+ cells (j) CD45 high CD11b+ TREM2+ cells (k) CD45 high CD11b+ CD206+ cells as a percentage of CD45+ cells. Data are shown as mean ± SEM. one-way ANOVA, *p<0.05, **p<0.01, ***p<0.001. l. Scheme illustrating the experimental design - mice were treated with either LPS_High or LPS_Low_Repeated or LPS_Low or PBS, prefrontal cortex including hippocampus were then dissected. Flow assisted cell sorting (FACS) was done on the single-cell suspensions followed by RNA-sequencing (n = 4 (males) in each group). m. FACS strategy for the sorting of CD45 low-mid CD11b+ cells, CD45 high CD11b+ cells, CD45− ACSA2+ cells respectively. n-t. RT-qPCR analysis of FACS sorted populations expressing typical cell-type specific markers, (n-r) CD45 low-mid CD11b+ and CD45 high CD11b+ sorting enriched the cells that express microglial markers exclusively (s-t) CD45− ACSA2+ sorting enriched the cells that express astrocyte markers exclusively.

To further elucidate the effects of repeated low-dose LPS treatment on glial cell populations within the forebrain and to identify the cellular subsets involved, we performed flow cytometry analyses using key surface markers that distinguish myeloid cell subsets based on CD45 and CD11b expression (Figure 3a). Cells were initially gated based on CD45 and CD11b expression which identified two distinct populations i.e. CD45^low-mid^ CD11b^+^ and CD45^high^ CD11b^+.^ This was followed by further characterization using TMEM119, F4/80, TREM2, and CD206 markers (Supp fig. 3a) to assess their activation states. Quantitative analysis revealed that the frequency of CD45^low-mid^ CD11b^+^ cells remained unchanged significantly in LPS_High, and LPS_Low_Repeated groups compared to PBS (Figs. 3a and 3b). However, the CD45^high^ CD11b^+^ population exhibited a significant increase in the LPS_Low_Repeated group compared to PBS (Figs. 3a and 3c). Further marker analysis within the CD45^low-mid^ CD11b^+^ subset showed a decreasing trend of TMEM119^+^ cells (Fig. 3d), indicating that the identity of these cells as resident microglia is affected. However, there was a significant elevation of F4/80^+^ cells following LPS_Low_Repeated treatment (Fig. 3e), suggesting an activation in this microglial subset. Interestingly, there was a notable decrease in TREM2^+^ cells in both LPS groups (Fig. 3f). Additionally, CD206^+^ cells were depleted in the LPS_High group but remained unaffected in the LPS_Low_Repeated compared to PBS (Fig. 3g).

In stark contrast to that of the CD45^low-mid^ CD11b^+^ subset, marker analysis within the CD45^high^ CD11b^+^ subset revealed a significant increase in TMEM119^+^ cells (Fig. 3h), suggesting a microglia-like / CNS-resident–biased identity. Additionally, there were marked increases in both F4/80^+^ (Fig. 3i) and TREM2^+^ cells (Fig. 3j) within the CD45^high^ CD11b^+^ population following LPS_Low_Repeated treatment. Assessment of CD206, marker commonly associated with phagocytic and endocytic myeloid states, demonstrated significant enrichment of CD206^+^ cells in this population (Fig. 3k), consistent with a shift towards phagocytic phenotype after repeated low-dose LPS treatment.

Next, to further investigate the transcriptional profiles of the two distinct CD45 populations affected by the varying LPS regimens, we performed flow-assisted cell sorting (FACS) to collect the CD45^low-mid^ CD11b^+^ and CD45^high^ CD11b^+^ populations after the LPS treatments, followed by RNA-sequencing. Additionally, we also sorted CD45^−^ ACSA2^+^ cells to study the LPS regimens’ effects on astrocytes (Fig. 3l). Mice were treated with either LPS_High, LPS_Low, LPS_Low_Repeated, or PBS as a control. We also included a single LPS low dose treatment in order to compare its’ effects alongside the other regimens and these analyses are listed in the supplementary data (Supp figs. 4a-z, Supp figs. 5a-e, Supp figs. 6a and 6b). The prefrontal cortex, including the hippocampus, was dissected, and single-cell suspensions were prepared for FACS (Fig. 3m). Post-sorting, we validated the purity of the isolated cell populations using qPCR to assess the expression of cell-type specific marker genes (Figs. 3n-3t). Cell-type specific transcript expression analysis demonstrated that both CD45 ^low-mid^ CD11b^+^ and CD45 ^high^ CD11b^+^ sorted cells were highly enriched for microglial marker genes (Figs. 3n-3r). These cells exhibited exclusive expression of microglial-specific markers, with no significant expression detected in CD45^−^ ACSA2^+^ cells. Conversely, the CD45^−^ ACSA2^+^ sorted cells showed exclusive enrichment of astrocyte-specific marker genes (Figs. 3s and 3t). These findings validate the specificity of our FACS sorting strategy, ensuring that the subsequent RNA-seq analyses accurately capture and reflect the transcriptional profiles of the targeted glial cell populations.

### Figure 4:

**Figure 4.**
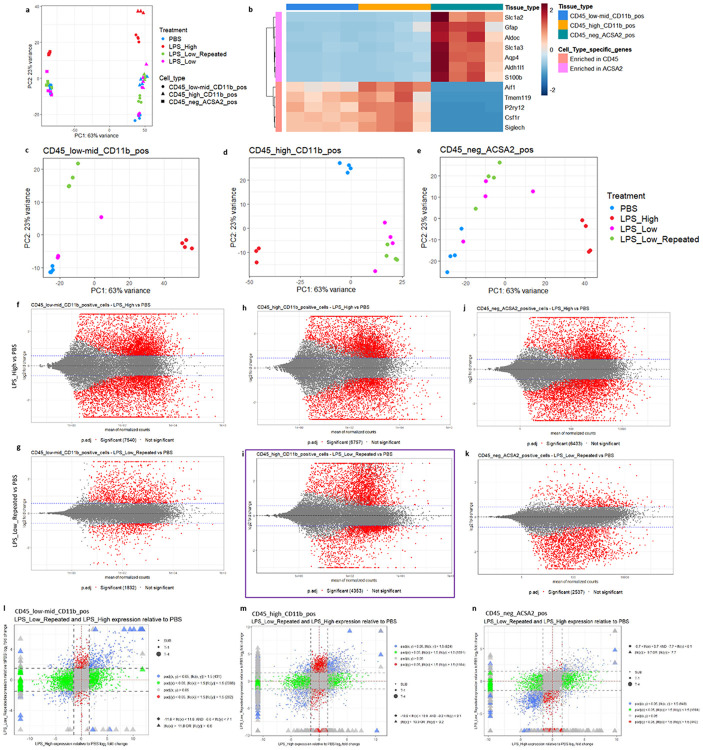
Cell-type–specific transcriptomics uncover distinct and restrained responses to repeated low-dose LPS across glial populations. a. PCA plot showing the global transcriptomic differences across treatments PBS, LPS_High, LPS_Low, and LPS_Low_Repeated in the cell types CD45_low-mid_CD11b_pos, CD45_high_CD11b_pos, and CD45_neg_ACSA2_pos. Different colors represent different treatment groups, and different shapes represent distinct cell types. b. Heatmap showing the enrichment of selected cell type-specific genes across the three defined cell populations. Color intensity represents scaled expression levels, with red indicating higher expression and blue indicating lower expression. The dendrogram on the left clusters the genes based on similarity, and the color bars above the heatmap indicate the corresponding cell types. c-e. Separate PCA plots for individual cell populations (c) CD45_low-mid_CD11b_pos, (d) CD45_high_CD11b_pos, and (e) CD45_neg_ACSA2_pos. Each plot highlights treatment-specific differences within each cell type, with treatments marked by colors (PBS in blue, LPS_High in red, LPS_Low in pink, and LPS_Low_Repeated in green), and the variance explained by PC1 and PC2 shown on the axes. f-k. MA plots of the pairwise comparisons of LPS treatments in the three cell types for LPS treatments LPS_High and LPS_Low_Repeated as compared to PBS; log fold changes (LFCs) are plotted against the mean of normalized counts to determine the variance between two treatments in terms of gene expression. Red nodes on the graph represent statistically significant data points i.e. p.adj < 0.05 and LFC > 1.5. Gray nodes are data points that are not statistically significant. Numerical values in parentheses for the significant legend indicate the number of genes that meet the prior condition. Dashed lines indicate the cutoff LFC values. (f) 7540 genes are significantly differentially expressed in CD45_low-mid_CD11b_positive_cells in the LPS_High versus PBS comparison. (g) 1832 genes are significantly differentially expressed in CD45_low-mid_CD11b_positive_cells in the LPS_Low_Repeated versus PBS comparison. (h) 6757 genes are significantly differentially expressed in CD45_high_CD11b_positive_cells in the LPS_High versus PBS comparison. (i) 4353 genes are significantly differentially expressed in CD45_high_CD11b_positive_cells in the LPS_Low_Repeated versus PBS comparison. (j) 6433 genes are significantly differentially expressed in CD45_neg_ACSA2_positive_cells in the LPS_High versus PBS comparison. (k) 2537 genes are significantly differentially expressed in CD45_neg_ACSA2_positive_cells in the LPS_Low_Repeated versus PBS comparison. l. Scatter plot depicting gene expression changes in CD45_low-mid_CD11b_pos cells comparing LPS_Low_Repeated and LPS_High treatments relative to PBS. The x-axis shows the log fold change (LFC) for LPS_High versus PBS, while the y-axis shows the LFC for LPS_Low_Repeated versus PBS. Colored dots represent genes categorized based on their significance (adjusted p-value < 0.05) and fold change thresholds (LFC > 1.5). Blue dots (431 genes) are significant in both comparisons, with LFC > 1.5 in either direction. Green dots (2386 genes) are significant in one comparison with LFC > 1.5, but not significant in the other. Red dots (262 genes) are significant in both comparisons, but with LFC < 1.5 in both directions. Gray dots are non-significant genes (adjusted p-value > 0.05). Shapes represent genes with extreme LFC values. Triangles represent genes with LFC beyond ±6.6, marking extreme upregulation or downregulation. Circles represent genes with more moderate LFC values. Dashed lines indicate the thresholds for fold change (LFC > 1.5), and red dashed lines represent the limits of the y-axis (LPS_Low_Repeated) LFC range between −11.8 and 11.8. m. Scatter plot depicting gene expression changes in CD45_high_CD11b_pos cells comparing LPS_Low_Repeated and LPS_High treatments relative to PBS. The x-axis shows the LFC for LPS_High versus PBS, while the y-axis shows the LFC for LPS_Low_Repeated versus PBS. Colored dots represent genes categorized based on their significance (adjusted p-value < 0.05) and fold change thresholds (LFC > 1.5). Blue dots (824 genes) are significant in both comparisons, with LFC > 1.5 in either direction. Green dots (1891 genes) are significant in one comparison with LFC > 1.5, but not significant in the other. Red dots (1184 genes) are significant in both comparisons, but with LFC < 1.5 in both directions. Gray dots are non-significant genes (adjusted p-value > 0.05). Shapes represent genes with extreme LFC values. Triangles represent genes with LFC beyond ±9.2, marking extreme upregulation or downregulation. Circles represent genes with more moderate LFC values. Dashed lines indicate the thresholds for fold change (LFC > 1.5), and red dashed lines represent the limits of the y-axis (LPS_Low_Repeated) LFC range between −10.9 and 10.9. n. Scatter plot depicting gene expression changes in CD45_neg_ACSA2_pos cells comparing LPS_Low_Repeated and LPS_High treatments relative to PBS. The x-axis shows the LFC for LPS_High versus PBS, while the y-axis shows the LFC for LPS_Low_Repeated versus PBS. Colored dots represent genes categorized based on their significance (adjusted p-value < 0.05) and fold change thresholds (LFC > 1.5). Blue dots (949 genes) are significant in both comparisons, with LFC > 1.5 in either direction. Green dots (1698 genes) are significant in one comparison with LFC > 1.5, but not significant in the other. Red dots (443 genes) are significant in both comparisons, but with LFC < 1.5 in both directions. Gray dots are non-significant genes (adjusted p-value > 0.05). Shapes represent genes with extreme LFC values. Triangles represent genes with LFC beyond ±7.7, marking extreme upregulation or downregulation. Circles represent genes with more moderate LFC values. Dashed lines indicate the thresholds for fold change (LFC > 1.5), and red dashed lines represent the limits of the y-axis (LPS_Low_Repeated) LFC range between −9.7 and 9.7. This plot highlights transcriptional differences in CD45_neg_ACSA2_pos cells across the two LPS treatments relative to the PBS.

PCA of the cell-type specific RNA-seq data revealed clear separation among treatments and cell types, indicating distinct transcriptional profiles induced by the various LPS regimens in each of the glial cell populations (Fig. 4a). The PCA plots for individual cell populations showed treatment-specific clustering within each cell type (Figs. 4c-e), suggesting that both microglia and astrocytes respond differently to LPS_High and LPS_Low_Repeated treatments. Scatterplot matrices comparing gene expression across treatments in each cell population showed overall expression patterns and variance between treatments (Supp figs. 4a–c). Additional PCA analyses confirmed the separation between whole-brain samples and isolated cell populations (Supp fig. 4d) and within the isolated cell populations themselves (Supp fig. 4e), also validating our sorting and sequencing approaches. A heatmap displaying the expression of selected cell-type specific genes further confirmed the purity of our sorted populations, with markers enriched in their respective cell types (Fig. 4b).

Differential gene expression analysis identified significant changes in gene expression induced by the various LPS treatments compared to PBS controls. In the CD45^low-mid^ CD11b^+^ microglia, LPS_High induced a large number of differentially expressed genes (DEGs), with 7,540 significant DEGs, whereas LPS_Low_Repeated resulted in 1,832 significant DEGs (Fig. 4f-g). Similarly, in the CD45^high^ CD11b^+^ population, LPS_High and LPS_Low_Repeated treatments led to 6,757 and 4,353 significant DEGs, respectively (Fig. 4h-i). This suggests that the repeated low dose LPS affects CD45^high^ CD11b^+^ population much more strongly than CD45^low-mid^ CD11b^+^ population. In the astrocyte population (CD45^−^ ACSA2^+^), LPS_High induced 6,433 significant DEGs, whereas LPS_Low_Repeated resulted in 2,537 significant DEGs (Fig. 4j-k). To compare and visualize the DEGs between LPS_High and LPS_Low_Repeated treatments relative to PBS, we generated two-way scatter plots for each cell type (Fig. 4l-n). Venn diagrams and upset plots illustrated the overlap of significant DEGs across the different LPS treatments within each cell type (Supp fig. 4i-n) and between the three cell types within each treatment (Supp fig. 4u–z), highlighting both shared and unique transcriptional responses.

### Figure 5:

**Figure 5.**
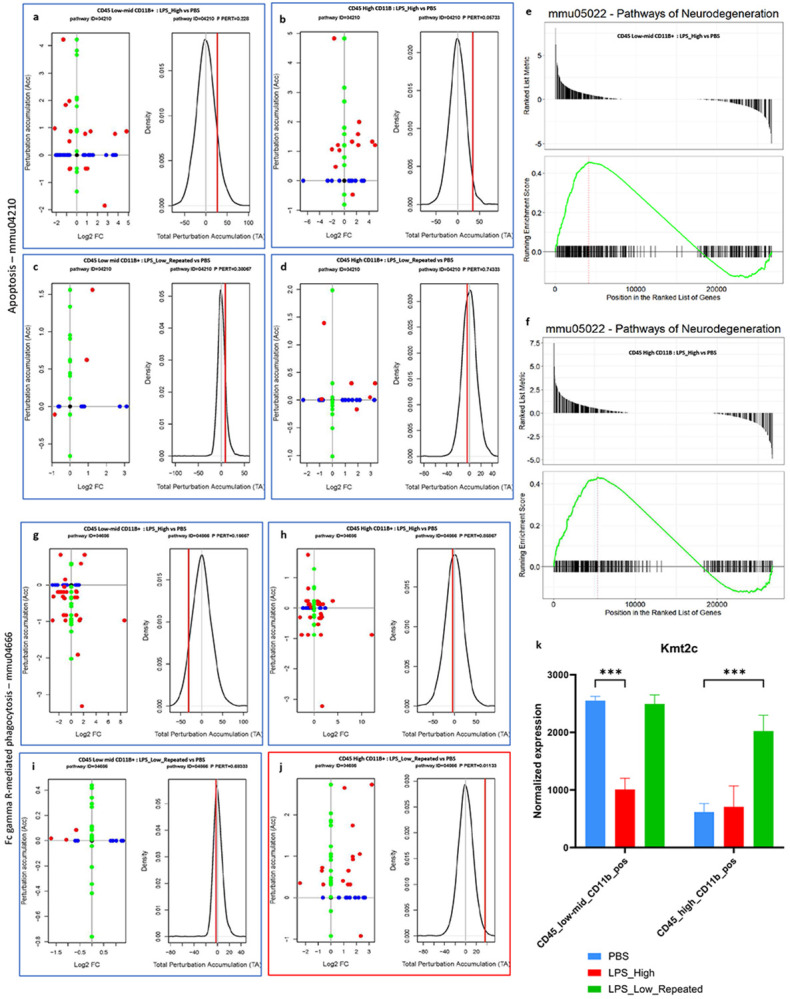
Repeated low-dose LPS enhances FcγR-mediated phagocytosis in CD45 ^high^ CD11b^+^ population a-d. Perturbation plots for the apoptosis pathway (KEGG ID mmu:04210) (a) in CD45_low-mid_CD11b_pos cells comparing LPS_High vs PBS (b) in CD45_high_CD11b_pos cells comparing LPS_High vs PBS (c) in CD45_low-mid_CD11b_pos cells comparing LPS_Low_Repeated vs PBS, and (d) in CD45_high_CD11b_pos cells comparing LPS_Low_Repeated vs PBS. The perturbation of all genes in the pathway are depicted as a function of the log2 fold changes (left panel). Non differentially expressed genes are assigned 0 log2 fold-change. The null distribution of the net accumulated perturbation is also shown as a grey vertical line (right panel). The observed total accumulation (tA) with the actual data is shown as a red vertical line (right panel). e-f. Gene Set Enrichment Analysis (GSEA) plots for the neurodegeneration pathway (KEGG ID mmu:05022). The ranked list of genes is plotted for each comparison. (e) GSEA plot for CD45_low-mid_CD11b_pos cells comparing LPS_High vs PBS. (f) GSEA plot for CD45_high_CD11b_pos cells comparing LPS_High vs PBS. The top panel in each plot shows the ranked list metric, while the bottom panel shows the running enrichment score (green line) for the neurodegeneration pathway across the ranked gene list. The position of pathway-related genes in the ranked list is indicated by vertical lines. g-j. Perturbation plots for the Fc gamma R-mediated phagocytosis pathway (KEGG ID mmu:04666) (g) in CD45_low-mid_CD11b_pos cells comparing LPS_High vs PBS (h) in CD45_high_CD11b_pos cells comparing LPS_High vs PBS (i) in CD45_low-mid_CD11b_pos cells comparing LPS_Low_Repeated vs PBS, and (j) in CD45_high_CD11b_pos cells comparing LPS_Low_Repeated vs PBS. The perturbation of all genes in the pathway are depicted as a function of the log2 fold changes (left panel). Non differentially expressed genes are assigned 0 log2 fold-change. The null distribution of the net accumulated perturbation is also shown as a grey vertical line (right panel). The observed total accumulation (tA) with the actual data is shown as a red vertical line (right panel). k. Bar plot showing the normalized expression levels of Kmt2c in CD45_low-mid_CD11b_pos and CD45_high_CD11b_pos cells across three treatment groups: PBS (blue), LPS_High (red), and LPS_Low_Repeated (green).

We next performed functional analyses on the DEG sets of CD45^low-mid^ CD11b^+^and CD45^high^ CD11b^+^ populations to investigate the signaling pathways modulated by different LPS treatments in the two myeloid populations, using SPIA and GSEA. In the LPS_High group, there was significant perturbation of the apoptosis pathway (KEGG ID mmu04210) in both CD45^low-mid^ CD11b^+^ microglia (Fig. 5a) and CD45^high^ CD11b^+^cells (Fig. 5b), as indicated by the total accumulation (tA) suggesting enhanced pro-apoptotic signaling. In contrast, the LPS_Low_Repeated treatment did not significantly perturb the apoptosis pathway in either cell populations (Figs. 5c and 5d). LPS_High treatment also resulted in enrichment of the neurodegeneration pathway (KEGG ID mmu05022) in both CD45^low-mid^ CD11b^+^ microglia (Fig. 5e) and CD45^high^ CD11b^+^ cells (Fig. 5f). In contrast, the LPS_Low_Repeated group did not exhibit significant enrichment of the neurodegeneration pathway in either cell populations. Robust activation of NF-κB signaling (KEGG ID mmu04064) was observed in both CD45^low-mid^ CD11b^+^ microglia and CD45^high^ CD11b^+^ cells (Supp fig. 5f and 5g), consistent with a pro-inflammatory profile after LPS_High treatment. However, the LPS_Low_Repeated treatment showed limited activation in both cell populations (Supp figs. 5h and 5i), indicating a limited yet resolving inflammatory response. The LPS_High treatment led to a strong perturbation of the Fc gamma receptor-mediated phagocytosis pathway (KEGG ID mmu04666) in both CD45^low-mid^ CD11b^+^ microglia (Fig. 5g) and CD45^high^ CD11b^+^ cells (Fig. 5h). Interestingly, the LPS_Low_Repeated treatment exclusively enriched the phagocytosis pathway in CD45^high^ CD11b^+^ cells (Figs. 5i and 5j), while CD45^low-mid^ CD11b^+^ microglia showed no perturbation (Fig. 5i). Additionally, expression analysis revealed that *Kmt2c*, a gene involved in immune tolerance-mediated neuroprotection through histone methyltransferase activity(10), was upregulated exclusively in CD45^high^ CD11b^+^ cells after LPS_Low_Repeated treatment. (Fig. 5k) and not in CD45^low-mid^ CD11b^+^ microglia population.

These findings indicate that while high-dose LPS (LPS_High) induces pro-apoptotic and pro-inflammatory signaling pathways in both glial cell populations, the repeated low-dose regimen (LPS_Low_Repeated) selectively enhances phagocytic function in CD45^high^ CD11b^+^ cells without triggering apoptotic or inflammatory responses.

### Figure 6:

**Figure 6.**
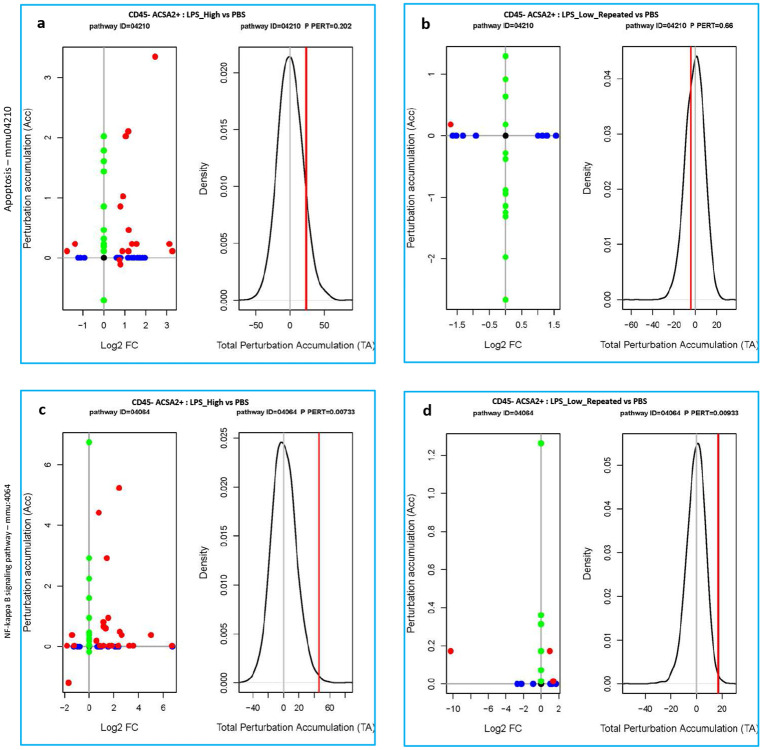
Astrocytes remain homeostatic under repeated low-dose LPS. a-b. Perturbation plots for the apoptosis pathway (KEGG ID mmu:04210) (a) in CD45_neg_ACSA2_pos cells comparing LPS_High vs PBS (b) in CD45_neg_ACSA2_pos cells comparing LPS_Low_Repeated vs PBS. c-d. Perturbation plots for the NF-kappa B signaling pathway (KEGG ID mmu:04064) (c) in CD45_neg_ACSA2_pos cells comparing LPS_High vs PBS (d) in CD45_neg_ACSA2_pos cells comparing LPS_Low_Repeated vs PBS. The perturbation of all genes in the pathway are depicted as a function of the log2 fold changes (left panel). Non differentially expressed genes are assigned 0 log2 fold-change. The null distribution of the net accumulated perturbation is also shown as a grey vertical line (right panel). The observed total accumulation (tA) with the actual data is shown as a red vertical line (right panel).

In CD45^−^ ACSA2^+^ astrocytes, SPIA revealed a strong activation of the apoptosis pathway in LPS_High-treated astrocytes (Fig. 6a), while LPS_Low_Repeated-treated astrocytes exhibited none (Fig. 6b). Similarly, LPS_High treatment led to significant perturbation of the NF-κB pathway (Fig. 6c). In contrast, LPS_Low_Repeated did not induce NF-κB pathway activation (Fig. 6d). Additionally, we did not observe the expression of neurotoxic A1 astrocyte-related genes following LPS_Low_Repeated treatment (Supp fig. 6c), in contrast to the upregulation seen in the LPS_High treatment.

We further assessed the effects of LPS_Low_Repeated treatment on the body weight. The body weight of mice treated with either PBS or LPS_Low_Repeated was monitored across 5 days of treatment and again at day 90 post-treatment (Supplementary Fig. 6d). Though the mice treated with repeated low-dose LPS showed a slight reduction in the body weight initially, they began to recover by day 3. No significant long-term differences were observed between the treatments, indicating that repeated low-dose LPS treatment did not result in lasting body weight changes. We further tested whether LPS_Low_Repeated treatment affected the progression of cognitive decline in a PS19 transgenic model, a widely used mouse model that overexpresses mutant tau and recapitulates key aspects of tauopathy, including neurodegeneration and memory deficits. Cognitive function was evaluated using the Morris Water Maze (MWM) behavior test, which measures spatial memory retention (Supp figs. 6e-h). During the hidden platform training phase of the MWM (Supp fig. 6e), PS19 Non-Tg PBS mice demonstrated consistent improvements in escape latency over the 7-day training period, indicating effective learning. In contrast, PS19 Tg PBS mice exhibited significantly longer escape latencies, confirming cognitive impairment. Interestingly, PS19 Non-Tg mice treated with LPS_Low_Repeated performed similarly to the PS19 Non-Tg PBS group, with no observable learning impairments. PS19 Non-Tg mice treated with PBS spent significantly more time in the target quadrant compared to PS19 Tg PBS mice, demonstrating impaired memory in the transgenic group. PS19 Non-Tg mice that received LPS_Low_Repeated showed similar performance to the PS19 Non-Tg PBS group, indicating that the repeated low-dose LPS treatment did not impair spatial memory in non-transgenic mice. However, while PS19 Tg mice treated with LPS_Low_Repeated showed only a modest improvement over the PS19 Tg PBS group, the difference was not statistically significant.

Collectively, across bulk tissue, flow cytometry, and cell-type–resolved transcriptomics, LPS_Low_Repeated diverges sharply from LPS_High by preserving glial homeostasis and selectively expanding a phagocytosis-competent CD45^high^ CD11b^+^ compartment, thereby establishing a tolerant, non-inflammatory CNS state.

## Discussion:

This study provides a comprehensive dissection of the CNS consequences of repeated low-dose LPS compared to single high-dose LPS, revealing fundamentally divergent glial responses. Repeated low-dose LPS induces transcriptional responses that diverge qualitatively from those elicited by acute high-dose exposure, consistent with differential innate immune conditioning rather than simple scaling of inflammatory intensity. Whereas LPS_High evoked a classical neuroinflammatory state characterized by cytokine induction, NF-κB activation, and apoptotic signaling (5, 6), LPS_Low_Repeated induced a tolerant yet functionally primed state that preserved glial homeostasis and enhanced phagocytic activity. These results extend prior work on innate immune memory in the brain(10, 12) by identifying CD45^high^ CD11b^+^ microglia as key effectors of immune tolerance with potential neuroprotective functions.

LPS_High triggered robust upregulation of *Il6, Tnf, Il1a, and Il1b* and suppressed homeostatic microglial genes such as *P2ry12 and Fcrls*, consistent with prior studies demonstrating acute neuroinflammation (5, 6). In contrast, LPS_Low_Repeated preserved homeostatic genes and enhanced phagocytic markers including *Clec7a, Cx3cr1, and Mrc1*, suggesting a reorientation of microglia toward clearance functions. Importantly, immunostaining revealed increased IBA1 as well as F4/80 expression more markedly after LPS_Low_Repeated regimen, and to some extent after LPS_High dosing. While under high-dose conditions this accompanied inflammatory cytokine induction, in the repeated low-dose paradigm, microglial IBA1 and F4/80 increase as well as astrocytic GFAP increase occurred without evidence of heightened inflammatory signaling, pointing toward a distinct, protective activation state. Importantly, increases in canonical “reactivity” markers such as IBA1 (AIF1) and GFAP should not be interpreted as synonymous with a pro-inflammatory state. Glial reactivity is multidimensional and context dependent, and can reflect structural remodeling, phagocytic engagement, or adaptive immune conditioning that does not map linearly onto cytokine induction or NF-κB activation. Our data demonstrate that under repeated low-dose LPS exposure, elevated IBA1/F4/80 signal occurs in the absence of inflammatory transcriptional amplification, supporting a tolerance-associated remodeling program rather than classical neuroinflammation.

These results emphasize that glial ‘activation’ cannot be inferred solely from marker intensity, as increased IBA1, F4/80, or GFAP may arise in either inflammatory or protective contexts, and importantly under LPS_Low_Repeated, these changes reflect a homeostatic, clearance-oriented phenotype rather than neurotoxic activation(14). The decoupling of glial activation from pro-inflammatory cytokine output suggests that repeated low-dose LPS engages protective glial program, distinct from the neurotoxic effects of high-dose exposure.

Whole-forebrain transcriptomics further confirmed the qualitative divergence between dosing regimens. LPS_High induced widespread differential expression and strong enrichment of NF-κB and apoptotic pathways, whereas LPS_Low_Repeated resulted in far fewer DEGs, enriched in phagocytic and amyloid clearance processes. Such selective retention of phagocytic pathway may represent an adaptive remodeling that enables long-term neuroprotection.

A distinct feature of this CNS tolerant state is the expansion of a CD45^high^ CD11b^+^ microglial population. These cells expressed TMEM119, supporting their resident microglia-like identity rather than infiltrating monocytes, in line with preserved blood–brain barrier integrity under low-dose regimens(10, 32). Importantly, this cell population showed elevated *Trem2, F4/80,* and CD206 expression—markers of an active, phagocytosis-competent state. This phenotype resonates with prior descriptions of CD45^high^ microglia as a subset with enhanced phagocytic activity (19), and further uncovers that such cells preferentially expand under conditions of CNS immune tolerance. The induction of F4/80 across both histological and flow-based readouts, but only accompanied by inflammatory cytokines in the LPS_High condition, further emphasizes that identical markers can represent divergent functional states depending on the immune milieu.

The transcriptional landscape of these CD45^high^ CD11b^+^ cells revealed selective enrichment of FcγR-mediated phagocytosis pathways, while apoptotic and neurodegenerative signatures were conspicuously absent. Notably, *Kmt2c*, a histone methyltransferase linked to H3K4me1-mediated immune tolerance, was upregulated in this subset, suggesting epigenetic remodeling as a basis for their stable functional shift(10). TMEM119, beyond its use as a microglial marker(33), it has recently been shown to bind amyloid-β and facilitate its clearance via LRP1 recruitment, restoring homeostasis and cognitive performance in mouse models(34). The preservation of TMEM119 in our CD45^high^ cells therefore underscores their potential relevance for disease contexts where amyloid burden drives pathology.

Astrocytic responses paralleled these findings. Under LPS_High, astrocytes activated NF-κB and apoptotic programs and upregulated neurotoxic A1 genes, consistent with maladaptive glial states seen in inflammatory neurodegeneration(20, 35). In contrast, astrocytes exposed to LPS_Low_Repeated had preserved homeostatic gene expression and avoided induction of apoptotic pathways. Behavioral assays further confirmed that repeated low-dose treatment did not impair spatial memory. These findings suggest that tolerance reshapes both microglial and astrocytic compartments to support homeostasis rather than drive injury, reinforcing the view that repeated low-dose systemic stimuli can promote CNS resilience.

From a translational perspective, harnessing the CD45^high^ CD11b^+^ microglia that emerge under tolerance could offer a means to enhance phagocytic clearance in neurodegenerative disease, where impaired debris removal is a key pathogenic feature. However, it is unknown how long these cells persist in the CNS beyond 9 months(10) and whether their phagocytic activity is sustained till then. While LPS itself cannot be applied in humans due to its systemic toxicity and risk of endotoxemia, the principle of inducing this phenotype remains an option. Thus, while our data underscore the promise of resident CD45^high^ microglia as therapeutic mediators, further work is needed to define their stability, mechanisms, and feasibility for translation.

This study delineates how distinct systemic LPS dosing regimens shape CNS glial states at phenotypic and transcriptional resolution. Pathways and regulators identified here, including phagocytosis-related programs and epigenetic modifiers such as *Kmt2c*, are presented as features of tolerance-associated glial states rather than as causally validated drivers, and their persistence and functional significance will require future investigation.

In sum, this work shows that dosing strategy determines whether systemic LPS exposure drives destructive neuroinflammation or fosters a tolerant state that preserves glial homeostasis. Repeated low-dose treatment selectively expands a population of resident CD45^high^ CD11b^+^ microglia that adopt a phagocytosis-competent but non-inflammatory profile, while astrocytes remain free of neurotoxic signatures. These findings provide a cellular and molecular framework for understanding how peripheral immune signals can reshape the CNS in protective ways. Looking ahead, the challenge will be to determine how such glial states can be harnessed to rebalance the inflammatory environment of the diseased brain during progressive neurodegeneration without compromising essential immune functions.

## Supplementary Material

This is a list of supplementary files associated with this preprint. Click to download.

- Supplementaryfiures01292026.pdf
- SupplementaryFigurelegends02102026.pdf
